# When the Skin Tells a Bigger Story: Distinguishing Cutaneous Metastases from Primary Adnexal Carcinomas in Dermatopathology

**DOI:** 10.3390/biology15141108

**Published:** 2026-07-09

**Authors:** Elsayed Ibrahim, Phyu P. Aung

**Affiliations:** Department of Anatomical Pathology, The University of Texas MD Anderson Cancer Center, 1515 Holcombe Blvd, Houston, TX 77030, USA

**Keywords:** cutaneous metastases, adnexal carcinoma, dermatopathology, immunohistochemistry, p63, calretinin, D2-40, cytokeratin 15, gene fusions, Muir–Torre syndrome, diagnostic algorithm

## Abstract

Cutaneous metastases are an uncommon but clinically significant manifestation of internal malignancy, occurring in approximately 0.7–10.4% of patients with cancer. In some cases, they represent the first clinical manifestation of an otherwise occult malignancy, making accurate diagnosis critical for timely patient management. Distinguishing cutaneous metastases from primary cutaneous neoplasms, particularly malignant adnexal tumors, remains one of the most challenging problems in dermatopathology because of their substantial clinical, histopathologic, and immunophenotypic overlap. This review summarizes the epidemiology and biology of cutaneous metastasis and the clinical presentation of cutaneous metastases and emphasizes the central role of clinicopathologic correlation in diagnosis. Histologic features such as a purely dermal or subcutaneous location, intravascular tumor emboli, and a “bottom-heavy” growth pattern favor metastasis, whereas the presence of an in situ component or transition from a benign precursor lesion strongly supports a primary cutaneous neoplasm. We review the diagnostic utility of optimized immunohistochemical panels, highlighting the complementary roles of p63, cytokeratin 15, calretinin, and D2-40 (podoplanin) in establishing primary adnexal lineage, together with emerging markers including SOX10, androgen receptor, TRPS1, adipophilin, INSM1, SATB2, and BerEP4 for diagnostically challenging cases. We also discuss recent advances in molecular biology and comprehensive genomic profiling, including recurrent gene fusions (e.g., *MYB::NFIB*, *CRTC1::MAML2*, and *YAP1* fusions) and characteristic mutational signatures that provide increasingly robust diagnostic evidence of tumor lineage. Finally, we provide a comprehensive, practical diagnostic algorithm for differentiating primary cutaneous adnexal carcinomas from cutaneous metastases of adenocarcinomas. By integrating traditional histopathologic techniques with modern immunohistochemical and molecular techniques, pathologists and clinicians can successfully navigate this complex differential diagnosis and thereby facilitate appropriate patient management and therapeutic intervention.

## Simple Summary

Cancer can sometimes spread from an internal organ to the skin, where it may closely resemble a skin cancer that started in the skin itself. Although these two conditions can appear nearly identical under the microscope, they require very different treatments and have different outcomes. This review explains the clinical features, microscopic findings, laboratory tests, and recent genetic discoveries that help doctors distinguish skin metastases from primary skin adnexal cancers, which arise from structures such as sweat glands, hair follicles, and sebaceous glands. We summarize both established and emerging diagnostic markers and describe how modern molecular testing can improve diagnostic accuracy in difficult cases. We also present a practical step-by-step diagnostic approach that integrates clinical information with microscopic examination, specialized laboratory tests, and molecular findings. By helping pathologists make the correct diagnosis more accurately and efficiently, this review supports timely treatment decisions, reduces the risk of misdiagnosis, and ultimately improves care for patients with cancer or rare skin tumors.

## 1. Introduction

Metastasis of internal malignant neoplasms to the skin is uncommon but clinically significant. Cutaneous metastases occur in approximately 0.7% to 10.4% of all patients with cancer and account for roughly 0.2% to 0.5% of all specimens evaluated in a typical dermatopathology practice [1,2]. While usually cutaneous metastases manifest in patients with a known history of widespread metastatic disease, in up to 17% of cases cutaneous metastases are the initial presenting sign of an occult internal malignant neoplasm [2,3]. The diagnosis of cutaneous metastases generally carries a poor prognosis, with median survival times often measured in months, underscoring the critical need for prompt and accurate identification [1].

One of the greatest diagnostic challenges in dermatopathology is distinguishing cutaneous metastases from primary cutaneous neoplasms, particularly malignant cutaneous adnexal tumors [4]. Primary adnexal carcinomas encompass eccrine, apocrine, follicular, and sebaceous lineages and exhibit a wide spectrum of morphologic patterns that can perfectly mimic cutaneous metastases from adenocarcinomas of the breast, lung, gastrointestinal tract, and kidney [4,5]. Because the clinical management, staging, and prognosis of a primary cutaneous neoplasm differ drastically from those of a metastatic visceral malignant neoplasm, distinguishing between the 2 entities is of paramount importance [1]. A misdiagnosis in either direction carries serious consequences: misidentifying a primary adnexal carcinoma as a metastasis may deny the patient a potentially curative surgical resection, while misidentifying a metastasis as a primary tumor may lead to inappropriate local treatment and delay in systemic workup.

In this narrative review, we explore the epidemiological patterns and clinical presentations that provide initial diagnostic clues to differentiating between cutaneous metastases and primary adnexal carcinomas. We then detail the key histopathologic features that favor a primary versus metastatic origin. We emphasize the critical role of immunohistochemistry (IHC), including the utility of markers such as p63, cytokeratin 15 (CK15), D2-40, and calretinin, in establishing adnexal lineage. We discuss recent advances in molecular pathology, such as the identification of recurrent gene fusions and mutational signatures, that have revolutionized the classification of adnexal tumors and provided new tools for resolving diagnostically ambiguous cases [6,7]. Finally, we offer a robust, practical algorithm for differentiating cutaneous metastases from primary adnexal carcinomas.

## 2. Literature Search Methodology

This narrative review was based on a comprehensive literature search of the PubMed/MEDLINE, Scopus, and Web of Science databases. Searches were conducted using combinations of the following terms: cutaneous metastases, skin metastasis, adnexal carcinoma, primary cutaneous carcinoma, immunohistochemistry AND skin AND metastasis, gene fusion AND adnexal tumor, and dermatopathology AND metastatic adenocarcinoma. The search included articles published from January 1990 through December 2025.

Eligible studies included peer-reviewed original research articles, case series, and review articles published in English that addressed the clinical, histopathologic, immunohistochemical, or molecular distinction between primary cutaneous adnexal neoplasms and cutaneous metastases. Case reports were included only when they provided unique diagnostic insights or described novel molecular or immunophenotypic findings. Editorials, letters without original data, conference abstracts, and non-English publications were excluded.

Key relevant publications were identified through manual review of the reference lists of selected articles. Given the narrative nature of this review, the literature was synthesized qualitatively, with emphasis on studies that have informed contemporary diagnostic practice and recent advances in immunohistochemistry and molecular pathology.

## 3. Biology of Metastasis

Understanding the biology of cutaneous metastasis provides important context for the diagnostic challenges encountered in dermatopathology. Metastasis is a highly inefficient yet ultimately lethal multistep process in which only a small fraction of disseminated tumor cells successfully establish clinically detectable secondary lesions. The metastatic cascade consists of local invasion, intravasation, survival within the circulation, extravasation, and colonization of a distant tissue microenvironment [8].

The initial stages of metastasis involve epithelial–mesenchymal transition, characterized by downregulation of epithelial adhesion molecules such as E-cadherin and increased expression of matrix metalloproteinases, enabling local invasion and stromal remodeling [9,10]. Tumor cells subsequently enter the vascular or lymphatic circulation through angiogenesis-associated vascular remodeling. During hematogenous dissemination, circulating tumor cells must survive shear stress, anoikis, and immune surveillance, often by forming platelet-coated microemboli that facilitate immune evasion and vascular arrest [8].

It is important to recognize that primary tumors are inherently heterogeneous, comprising multiple distinct clonal populations including invasive, metastatic, pluripotent, and supportive cell colonies [8]. Only selected tumor subclones possess the molecular characteristics necessary to complete the metastatic cascade. Consequently, metastatic lesions often represent a biologically selected subset of the primary tumor and may exhibit less morphologic and molecular heterogeneity than their primary counterparts. This clonal selection has important diagnostic implications, as metastatic deposits may display relatively uniform cytologic and immunophenotypic features despite marked heterogeneity in the primary neoplasm.

The propensity of certain malignancies to metastasize to the skin (dermotropism) reflects the principles of Paget’s classic “seed and soil” hypothesis [11]. The skin provides a unique microenvironment rich in blood vessels, lymphatics, extracellular matrix, and resident immune cells that can support metastatic colonization. Primary tumors actively establish premetastatic niches through the secretion of cytokines, growth factors, and extracellular vesicles that increase vascular permeability, remodel the extracellular matrix, and recruit immunosuppressive inflammatory cells [12]. In addition, chemokine receptor-ligand interactions (e.g., CXCR4–CXCL12) and integrin-mediated adhesion contribute to organ-specific homing of circulating tumor cells. These mechanisms help explain the predilection of certain malignancies, particularly breast carcinoma and melanoma, to metastasize to the skin.

A proposed mechanism contributing to the establishment of cutaneous metastases is micro-infarction induced by tumor emboli within dermal arterioles. Tumor cell embolization can occlude small dermal vessels, resulting in focal ischemia and tissue infarction. The ensuing ischemic injury elicits a localized inflammatory response characterized by neutrophil recruitment, release of pro-inflammatory cytokines, and increased vascular permeability [12]. This inflammatory microenvironment may paradoxically promote metastatic colonization by facilitating additional tumor cell extravasation, stimulating angiogenesis, and providing a supportive niche for tumor growth. This mechanism may also explain why some cutaneous metastases present with surrounding erythema and edema, clinically mimicking inflammatory dermatoses.

An understanding of these biological mechanisms also provides insight into the histopathologic features of cutaneous metastases. The absence of an in situ component, frequent localization within the dermis or subcutis, lymphovascular invasion, and preservation of the immunophenotypic profile of the primary tumor all reflect the biology of metastatic dissemination and form the basis of many of the diagnostic principles discussed throughout this review.

## 4. Epidemiology and Timing of Diagnosis of Cutaneous Metastases

The sites of origin of cutaneous metastases differ significantly by patient sex. In women, breast carcinoma is by far the most common source of cutaneous metastases, accounting for up to 70% of cases, followed by melanoma, ovarian cancer, and lung cancer [3,13]. In men, melanoma is the most frequent source of cutaneous metastases, accounting for approximately 32% of cases, followed by carcinomas of the head and neck (16%), lung (12%), and colon (11%) [3,13]. Cutaneous metastases in children are rare, and most are associated with rhabdomyosarcoma and neuroblastoma [3].

The temporal relationship between diagnosis of the primary tumor and diagnosis of cutaneous metastases is also diagnostically relevant. Approximately 60% of cutaneous metastases are diagnosed after the primary tumor, 32% are diagnosed at the same time as the primary tumor, and only 8% are diagnosed before the primary tumor [3]. Diagnosis of cutaneous metastases before the primary tumor, while uncommon, has been documented in prostate cancer, rectal carcinoma, and thyroid carcinoma, among others [3].

## 5. Morphology of Cutaneous Metastases

Clinically, cutaneous metastases exist along a wide morphologic spectrum. They most commonly present as firm, painless, rapidly growing nodules or plaques that are flesh-colored to erythematous [13]. However, they can also mimic a variety of benign or inflammatory dermatologic conditions. For instance, metastatic lesions may resemble lipomas, epidermoid cysts, or pyogenic granulomas [3]. Cutaneous metastases from inflammatory carcinoma (carcinoma erysipelatoides), most commonly from breast primary tumors, present as an erythematous, edematous plaque that closely mimics cellulitis due to extensive lymphatic permeation in the dermis [1]. The Sister Mary Joseph nodule, a periumbilical metastasis, is a classic presentation of an intra-abdominal or pelvic malignant neoplasm and should prompt a thorough systemic workup [1].

Dermoscopy can provide useful clinical clues to the origin of cutaneous neoplasms. Nonpigmented cutaneous metastases typically have highly vascularized structures with atypical vessels (dotted, irregular, or glomerular); pigmented patterns may be observed in metastases from melanoma or breast carcinoma [14]. Despite these clinical clues, the definitive distinction between a primary adnexal tumor and a cutaneous metastasis relies heavily on histopathologic and ancillary testing.

## 6. Interpretation of Histopathologic Findings in Cutaneous Neoplasms

The initial evaluation of a cutaneous neoplasm using routine hematoxylin and eosin staining remains the cornerstone of diagnosis. Several architectural and cytologic features can help guide the pathologist toward the diagnosis of a primary or metastatic tumor.

### 6.1. Features Favoring Metastasis

Metastatic cutaneous carcinomas typically present as well-circumscribed nodules centered within the deep reticular dermis and/or subcutaneous tissue, producing a characteristic “bottom-heavy” growth pattern [1]. They generally lack continuity with the overlying epidermis or native adnexal structures and do not exhibit an in situ component or transition from a benign precursor lesion, features that favor a primary cutaneous neoplasm [4]. Cytologically, metastatic tumors often appear “foreign” or incongruous relative to the surrounding cutaneous structures. Lymphovascular tumor emboli are a useful clue to metastatic disease, particularly when identified within dermal lymphatic channels. However, lymphovascular invasion is not specific and may also occur in aggressive primary cutaneous adnexal carcinomas, including hidradenocarcinoma and porocarcinoma. Accordingly, this finding should be interpreted in conjunction with the overall histopathologic and clinical context rather than as an isolated diagnostic criterion [1]. Certain cytomorphologic features should prompt consideration of a metastatic origin and may provide clues to the primary site. “Dirty” necrosis is characteristic of metastatic colorectal adenocarcinoma, whereas prominent signet ring cells raise suspicion for gastric adenocarcinoma or invasive lobular carcinoma of the breast. Clear cells associated with a delicate sinusoidal vascular network favor metastatic renal cell carcinoma, while finely granular (“salt-and-pepper”) chromatin with neuroendocrine cytologic features suggests metastatic small cell carcinoma or Merkel cell carcinoma [1,4].

### 6.2. Features Favoring Primary Adnexal Carcinoma

Primary cutaneous adnexal carcinomas frequently demonstrate continuity with the epidermis or native adnexal structures, including hair follicles, sebaceous glands, or sweat ducts [4]. The presence of an in situ component, such as intraepidermal Paget disease or porocarcinoma in situ, strongly supports a primary cutaneous neoplasm [1]. Likewise, identification of a morphologic transition from a benign precursor lesion (e.g., poroma to porocarcinoma) or a continuum of differentiation from well-formed adnexal structures to invasive carcinoma strongly favors a primary adnexal origin [4].

Although the absence of an epidermal or adnexal connection favors metastasis, this finding is not specific and should be interpreted with caution, particularly in superficial or limited biopsy specimens, poorly sampled tumors, or deeply invasive primary carcinomas in which the superficial component is not represented. Similarly, desmoplastic stroma and perineural invasion are characteristic features of certain primary adnexal carcinomas, particularly microcystic adnexal carcinoma, a locally aggressive neoplasm that rarely metastasizes despite exhibiting extensive perineural invasion in up to 80% of cases [15].

It is important to emphasize that the morphologic features discussed above are supportive rather than definitive. No single histopathologic feature is pathognomonic for either a primary cutaneous adnexal carcinoma or a cutaneous metastasis. Accurate diagnosis requires integration of clinical presentation, histopathologic findings, immunophenotype, and, when indicated, molecular studies. Ancillary testing should be performed whenever morphologic findings are equivocal or the diagnosis will influence patient management.

### 6.3. Morphologic Patterns Commonly Causing Confusion

Several specific morphologic patterns are notorious for causing confusion about whether cutaneous neoplasms are primary or metastatic (Table 1). Several of these patterns are discussed here; immunohistochemical markers used in such cases are discussed below in the section Immunohistochemical Panels.

Clear Cell Tumors. Primary cutaneous clear cell tumors, such as sebaceous carcinoma, clear cell hidradenoma, and clear cell porocarcinoma, can closely mimic metastatic clear cell RCC [4]. Distinguishing between primary clear cell tumors and metastatic RCC is particularly difficult because both RCC and some primary sebaceous tumors can express CD10 and EMA [1]. The identification of sebaceous lobules, comedonecrosis, or an epidermal connection favors a primary sebaceous neoplasm, while PAX8 positivity and CK7 negativity are characteristic of RCC [16].

Mucinous Tumors. Primary mucinous carcinoma of the skin is a rare adnexal tumor that is histologically indistinguishable from metastatic mucinous carcinoma of the breast or gastrointestinal tract [4]. Both present as islands of atypical epithelial cells floating in large pools of extracellular mucin. The presence of an in situ component within adnexal structures and the absence of a breast or gastrointestinal primary tumor are essential for establishing a primary cutaneous diagnosis.

Extramammary Paget Disease. Primary EMPD and secondary EMPD are particularly difficult to distinguish from one another. Primary EMPD arises as an intraepithelial neoplasm from pluripotent keratinocyte stem cells or adnexal glandular cells, while secondary EMPD results from the epidermotropic spread of an underlying adenocarcinoma (most commonly rectal or urothelial) [1]. The 2 forms share a pagetoid intraepidermal growth pattern with large cells containing abundant pale cytoplasm and prominent nucleoli. The distinction between primary and secondary EMPD is critical because the diagnosis of secondary EMPD mandates treatment of the underlying visceral malignant neoplasm.

In recent years, additional immunohistochemical markers have further improved the distinction between primary and secondary EMPD. TRPS1 (tricho-rhino-phalangeal syndrome type 1) has emerged as a sensitive marker for primary EMPD, demonstrating diffuse nuclear expression in most cases while typically remaining negative in secondary EMPD of colorectal origin [17]. HER2 overexpression is identified in a subset of primary EMPD and has important therapeutic implications, as affected patients may benefit from HER2-targeted therapy, including trastuzumab [17]. Uroplakin II and uroplakin III are highly specific markers of urothelial differentiation and are particularly useful for confirming secondary EMPD arising from urothelial carcinoma [17]. An important diagnostic consideration is the distinction between EMPD and pagetoid squamous cell carcinoma in situ (pagetoid Bowen disease). In contrast to Paget cells, pagetoid squamous lesions typically express p63, p40, and high-molecular-weight cytokeratins (e.g., CK5/6) while lacking CK7, GCDFP-15, and other markers of glandular differentiation. Accordingly, a carefully selected immunohistochemical panel, interpreted in conjunction with the clinical and histopathologic findings, is essential for establishing the correct diagnosis.

## 7. Immunohistochemical Panels

IHC is an indispensable adjunct in the differential diagnosis of cutaneous neoplasms. While no single marker is entirely specific for primary adnexal lineage, a carefully selected panel can significantly improve diagnostic accuracy.

### 7.1. Markers Favoring Primary Adnexal Carcinoma

The most widely studied panel for supporting a primary cutaneous adnexal carcinoma includes p63, CK15, D2-40 (podoplanin), and calretinin [18]. When a tumor exhibits positive staining for all or most of these markers, a primary cutaneous adnexal neoplasm is favored, although the diagnostic performance of this panel is imperfect and results must be interpreted in the appropriate clinicopathologic context [18].

p63 and p40. The p53 homologue p63 is expressed in the basal and myoepithelial cells of normal skin appendages and is highly sensitive for primary adnexal carcinomas; it is expressed in over 90% of primary adnexal carcinomas but only approximately 8% of metastatic adenocarcinomas [18]. However, it is important to recognize that p63 positivity can be observed in subsets of metastatic carcinomas, including squamous cell carcinomas of various origins, urothelial carcinomas, and occasional salivary gland tumors; therefore, p63 positivity alone does not exclude a metastatic origin [18,19]. The truncated isoform p40 (ΔNp63) has a sensitivity of approximately 84% and a specificity superior to that of p63 in distinguishing primary cutaneous adnexal carcinomas from cutaneous metastases [19].

Cytokeratin 15. CK15 is a marker of follicular stem cells located in the bulge region of the hair follicle. It is highly specific (up to 98%) for primary adnexal carcinomas, although its sensitivity is lower (approximately 40%) [18]. This limited sensitivity means that CK15 negativity does not exclude a primary adnexal origin, as CK15 is not universally expressed across all adnexal tumor subtypes and may be lost in poorly differentiated carcinomas. Its high specificity makes it a valuable confirmatory marker when positive.

D2-40 (Podoplanin). D2-40 is a transmembrane mucoprotein expressed in lymphatic endothelium, the basal cells of the epidermis, and the outer root sheath of hair follicles. D2-40 expression is seen in approximately 44% of primary adnexal carcinomas but is virtually absent in metastatic adenocarcinomas and has a specificity of approximately 96% for primary adnexal carcinomas [18,20]. The relatively low sensitivity of D2-40 (44%) represents a significant limitation, as more than half of primary adnexal carcinomas will be negative for this marker. Therefore, D2-40 negativity should not be used to exclude a primary adnexal origin.

Calretinin. Calretinin is a calcium-binding protein of the EF-hand family that is expressed in the innermost cell layer of the outer root sheath in normal anagen hair follicles, as well as in both the duct and secretory portions of eccrine sweat glands [21]. In the context of cutaneous neoplasms, calretinin is frequently positive in tumors with follicular differentiation (e.g., tricholemmoma, trichilemmal carcinoma, and pilomatricoma) and is positive in a significant proportion of sweat gland tumors [21,22]. Mahalingam et al. demonstrated calretinin expression in approximately 14% of primary adnexal carcinomas versus 10% of cutaneous metastases, suggesting a modest but real contribution to the panel [18]. However, other studies have reported calretinin positivity in up to 64% of cutaneous adnexal tumors, particularly those with follicular or eccrine differentiation [22]. This considerable variability in reported calretinin expression among different adnexal tumor types limits its reliability as an independent marker, and its contribution to the diagnostic panel should be considered supplementary rather than decisive. The inclusion of calretinin in the diagnostic panel, alongside p63, CK15, and D2-40, provides additional support for a primary cutaneous origin. An important caveat is that calretinin is also strongly expressed in malignant mesothelioma; therefore, in the rare scenario of calretinin positivity in combination with p63 negativity, cutaneous mesothelioma metastasis should be considered [23].

Limitations of the Four-Marker Panel. While the combination of p63, CK15, D2-40, and calretinin provides useful diagnostic information, several important limitations must be acknowledged. First, no single marker in this panel achieves both high sensitivity and high specificity simultaneously. Second, the reported diagnostic performance varies considerably across studies depending on the specific tumor subtypes included, antibody clones used, and scoring thresholds applied. Third, a negative result for all four markers is suggestive of but not diagnostic for metastasis, as poorly differentiated primary adnexal carcinomas may lose expression of these markers. The panel performs best when interpreted in conjunction with morphologic features and clinical context, and equivocal results should prompt consideration of additional markers or molecular testing.

### 7.2. Additional Immunohistochemical Markers in Contemporary Practice

Beyond the core four-marker panel, several additional immunohistochemical markers have expanded the diagnostic armamentarium for distinguishing primary cutaneous adnexal carcinomas from cutaneous metastases and for subclassifying specific adnexal neoplasms.

SOX10. SOX10 is a transcription factor involved in neural crest development and is expressed in melanocytes, Schwann cells, and myoepithelial cells. In cutaneous adnexal tumors, strong and diffuse nuclear SOX10 expression is characteristic of primary cutaneous adenoid cystic carcinoma and serves as a useful surrogate for *MYB::NFIB* fusion [6]. SOX10 is also expressed in tumors with myoepithelial differentiation, including mixed tumors (chondroid syringomas) and myoepitheliomas. Because SOX10 is also positive in metastatic melanoma and some salivary gland carcinomas, results should always be interpreted in the appropriate clinicopathologic context.

Androgen Receptor (AR). AR is frequently expressed in tumors with apocrine and sebaceous differentiation, including apocrine carcinoma and sebaceous neoplasms. However, AR is also commonly expressed in metastatic breast carcinoma (especially apocrine subtype) and salivary duct carcinoma. Therefore, AR positivity supports—but is not specific for—primary cutaneous apocrine or sebaceous differentiation and should be interpreted alongside other lineage-specific markers.

TRPS1. TRPS1 is a highly sensitive marker for breast carcinoma (>95% sensitivity) and is also expressed in primary extramammary Paget disease (EMPD). Consequently, TRPS1 positivity in a cutaneous adenocarcinoma should prompt consideration of metastatic breast carcinoma while recognizing primary EMPD as an important exception. Conversely, absent or reduced TRPS1 expression in secondary EMPD of colorectal origin may aid in distinguishing it from primary EMPD.

SATB2. SATB2 is a highly sensitive and specific marker of colorectal differentiation. In cutaneous tumors with glandular or mucinous morphology, SATB2 positivity, particularly when combined with CK20 and CDX2, strongly supports metastatic colorectal adenocarcinoma.

INSM1. INSM1 is a highly sensitive marker of neuroendocrine differentiation and is particularly useful for distinguishing metastatic small cell carcinoma and Merkel cell carcinoma from primary adnexal neoplasms with basaloid morphology. Compared with traditional neuroendocrine markers such as synaptophysin and chromogranin, INSM1 generally demonstrates superior sensitivity while maintaining excellent specificity.

Adipophilin. Adipophilin is a lipid droplet-associated protein that serves as one of the most sensitive markers of sebaceous differentiation. Its characteristic cytoplasmic vacuolar staining is particularly valuable for confirming sebaceous lineage in poorly differentiated carcinomas or highlighting subtle sebaceous differentiation not readily apparent on routine histology. In appropriate clinical settings, confirmation of sebaceous differentiation should also prompt consideration of Muir–Torre syndrome.

GATA3: Although GATA3 is a highly sensitive marker for breast carcinoma, it is also expressed in urothelial carcinoma, paraganglioma, and a subset of primary cutaneous adnexal tumors with apocrine differentiation. Accordingly, GATA3 positivity alone does not establish metastatic breast carcinoma and should be interpreted with additional markers such as ER, PR, mammaglobin, GCDFP-15, and TRPS1, together with the clinical context.

BerEP4 and EMA. BerEP4 (EpCAM) is widely used in dermatopathology as a marker of basal cell carcinoma but is also expressed in numerous primary and metastatic adenocarcinomas, limiting its discriminatory value. Similarly, epithelial membrane antigen (EMA) is broadly expressed in epithelial malignancies. Although neither marker reliably distinguishes primary from metastatic tumors, the pattern of EMA staining (luminal/apical versus diffuse) may provide clues regarding the direction of differentiation.

CK7/CK20. Most primary cutaneous adnexal carcinomas exhibit a CK7-positive/CK20-negative immunophenotype, a profile shared by breast and lung adenocarcinomas [1,4]. Consequently, a CK7+/CK20− profile cannot distinguish a primary adnexal carcinoma from metastatic breast or lung adenocarcinoma and should always be supplemented with lineage-specific markers. In contrast, a CK7−/CK20+ profile strongly argues against a primary cutaneous adnexal neoplasm and instead favors metastatic colorectal adenocarcinoma.

### 7.3. Markers for Metastases from Tumors in Specific Organs

When a metastatic origin is suspected on the basis of morphology or negative findings of a primary adnexal tumor panel, markers for tumors in specific organs are employed to identify the primary tumor site (Table 1).

Breast. Metastatic breast carcinoma is the most common source of cutaneous metastases in women. A panel including GATA3, estrogen receptor, progesterone receptor, and mammaglobin is highly sensitive and specific for breast carcinoma [24]. GATA3 is particularly useful as it is expressed in most breast carcinomas (sensitivity ~91%) but is typically negative in primary sweat gland carcinomas, though it may be expressed in urothelial carcinoma [24]. Loss of E-cadherin expression is characteristic of lobular breast carcinoma and its metastases and can help distinguish lobular breast carcinoma metastases from signet ring cell adnexal tumors [1].

It should be noted that triple-negative breast carcinomas may lack ER, PR, and GATA3 expression, making their distinction from primary adnexal carcinomas particularly challenging. In such cases, TRPS1 retains high sensitivity and may be the most reliable marker for confirming breast origin.

Lung. Metastatic lung adenocarcinoma typically expresses TTF-1, Napsin A, and CK7 [1]. The combination of TTF-1 and Napsin A positivity is highly specific for a pulmonary origin [25]. It should be noted that squamous cell carcinoma of the lung expresses p40 and CK5/6, markers that overlap with primary cutaneous squamous carcinoma and primary cutaneous adnexal carcinoma, necessitating careful clinical correlation.

Gastrointestinal Tract. Metastatic colorectal carcinoma is classically positive for CK20 and CDX2 and negative for CK7 [1]. The combination of “dirty necrosis” on hematoxylin and eosin staining and a CK20-positive, CDX2-positive, CK7-negative profile is virtually diagnostic of colorectal origin.

Kidney and Genitourinary Tract. Metastatic clear cell RCC is typically positive for PAX8 and CD10 and negative for CK7 and CK20 [1]. PAX8 is a highly sensitive and specific marker for tumors of renal, Müllerian (ovarian), or thyroid origin, making it invaluable in distinguishing metastases from such tumors from primary adnexal tumors [16]. However, it should be acknowledged that exceptions to the classic PAX8+/CD10+/CK7 immunophenotype of clear cell RCC exist; chromophobe RCC and papillary RCC may express CK7, and rare cases of clear cell RCC may show focal CK7 positivity. Therefore, a broader panel including carbonic anhydrase IX (CAIX) may be helpful in equivocal cases.

Extramammary Paget Disease. The IHC profile of EMPD is particularly instructive of the value of IHC in identifying tumor origin. Primary EMPD is typically CK7 positive, CK20 negative, TRPS1 positive, and GCDFP-15 positive, reflecting its apocrine glandular origin. Secondary EMPD associated with an anorectal malignant neoplasm typically exhibits the opposite staining pattern for these markers, i.e., CK7 negative, CK20 positive, TRPS1 negative, and GCDFP-15 negative, along with CDX2 positivity. Secondary EMPD associated with urothelial carcinoma typically is CK7 positive, CK20 positive, uroplakin positive, and GCDFP-15 negative. HER2 overexpression and/or amplification has been reported in up to 15–30% of primary EMPD cases and represents a potential therapeutic target [17]. Uroplakin II and III demonstrate high specificity for urothelial differentiation and should be included in the panel when secondary EMPD of urothelial origin is suspected.

## 8. Molecular Advances and Genomic Profiling of Cutaneous Adnexal Tumors

Recent advances in molecular pathology have revolutionized the classification of cutaneous adnexal tumors, revealing that many of these neoplasms harbor specific recurrent genetic alterations. These discoveries not only aid in resolving diagnostically challenging cases but also highlight the biological similarities between cutaneous adnexal tumors and homologous tumors in the salivary and mammary glands [6].

### 8.1. Recurrent Gene Fusions in Primary Adnexal Neoplasms

Several primary adnexal carcinomas are now defined by specific gene fusions, which can be detected via fluorescence in situ hybridization, RT-PCR, or next-generation sequencing. Table 2 summarizes the major molecular alterations in adnexal tumors.

Adenoid Cystic Carcinoma. Cutaneous adenoid cystic carcinoma is characterized by recurrent *MYB::NFIB* or *MYBL1::NFIB* gene fusions in up to 83% of cases, identical to its salivary gland counterpart [6]. MYB and SOX10 detected on IHC can serve as surrogate markers for fusion, with SOX10 showing intense and diffuse nuclear positivity in the vast majority of cases [6].

Poroma and Porocarcinoma. In poroma and porocarcinoma, *YAP1* fusions are present in a significant majority of cases and serve as strong diagnostic markers [6]. IHC for YAP1 C-terminus (showing cytoplasmic loss) and NUT (showing nuclear positivity) can be used as surrogate markers for the fusion in routine practice [6].

Hidradenoma and Hidradenocarcinoma. Hidradenoma and hidradenocarcinoma are characterized by *CRTC1::MAML2* or *CRTC3::MAML2* fusions, similar to mucoepidermoid carcinoma of the salivary gland [6]. This molecular overlap can cause diagnostic confusion when a *MAML2*-rearranged tumor presents in the skin as it may represent either a primary adnexal tumor or a metastasis from a salivary gland mucoepidermoid carcinoma.

Secretory Carcinoma. Cutaneous secretory carcinoma is defined by the *ETV6::NTRK3* gene fusion, which is also the hallmark of secretory carcinoma of the breast and salivary gland [6]. The identification of this fusion is diagnostically critical as it not only confirms the diagnosis but also identifies patients who may benefit from TRK inhibitor therapy (e.g., larotrectinib, entrectinib).

### 8.2. Sebaceous Neoplasms and Muir–Torre Syndrome

Sebaceous neoplasms are of special concern because they are associated with Muir–Torre syndrome, a variant of Lynch syndrome caused by germline mutations in mismatch repair (MMR) genes, most commonly *MSH2* and *MSH6* [26] (Table 2). IHC for MMR proteins (MLH1, PMS2, MSH2, and MSH6) should be performed on sebaceous neoplasms, especially from the non-head and neck area as loss of expression of MMR genes identifies patients who require genetic counseling and screening for synchronous or metachronous visceral malignant tumors, particularly colorectal, endometrial, and urothelial carcinomas [26]. Importantly, the presence of sebaceous differentiation in a carcinoma does not automatically indicate a primary cutaneous origin; metastatic carcinomas with sebaceous-like differentiation have been reported, and the clinical context must always be considered.

## 9. Comprehensive Genomic Profiling

Comprehensive genomic profiling has provided further insights into the pathogenesis of adnexal tumors. Sebaceous tumors exhibit the highest frequency of genomic alterations among adnexal carcinomas, including frequent mutations in RB1 (38.2%) and TP53 (76.4%), as well as microsatellite–high status in approximately 15.7% of cases [7]. In contrast, sweat gland tumors generally exhibit lower genomic alteration burdens, reflecting their distinct pathogenesis driven by specific gene fusions rather than widespread genomic instability [7]. The tumor mutational burden across most adnexal carcinoma types ranges from 10.4 mutations per megabase to 38.8 mutations per megabase [7]. Unlike basal cell and squamous cell carcinomas, which are driven by ultraviolet-induced mutational signatures, many deep-seated adnexal tumors lack ultraviolet signatures, further supporting their distinct biology [6,7].

Practical Considerations and Limitations of Molecular Testing. Although molecular alterations have emerged as powerful diagnostic biomarkers for cutaneous adnexal neoplasms, several practical considerations limit their routine implementation. Next-generation sequencing (NGS) panels capable of detecting gene fusions are not universally available and are largely confined to academic centers and specialized reference laboratories. Optimal detection of gene rearrangements often requires RNA-based sequencing, which may necessitate referral testing, increasing both cost and turnaround time (typically 2–4 weeks). In addition, comprehensive genomic profiling may not be reimbursed by all insurance providers, further limiting accessibility.

Accordingly, molecular testing is best regarded as a complementary diagnostic tool reserved for diagnostically challenging or clinically significant cases, rather than a first-line test for all cutaneous neoplasms. In routine practice, immunohistochemical surrogate markers provide a more accessible and cost-effective initial approach. Examples include SOX10 and MYB for adenoid cystic carcinoma and loss of YAP1 C-terminal expression for poroma and porocarcinoma. Confirmatory molecular testing can then be selectively employed when immunohistochemical findings are inconclusive or when definitive molecular characterization has therapeutic implications, such as identifying tumors eligible for TRK inhibitor therapy.

## 10. Practical Diagnostic Algorithm

**Figure 1:**
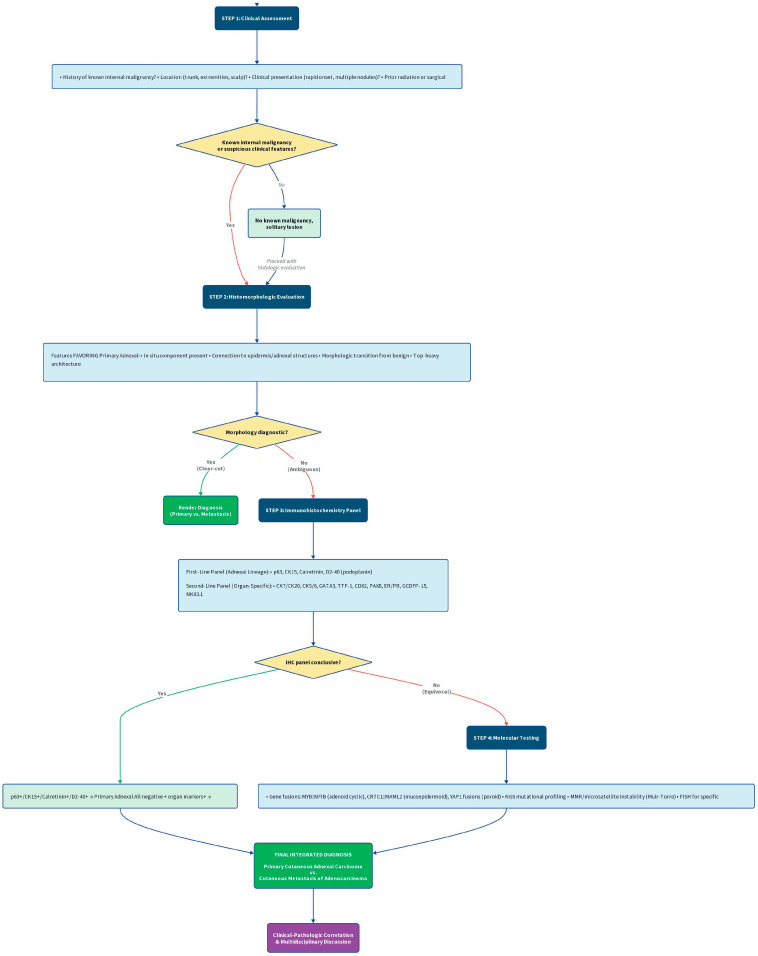
Proposed stepwise diagnostic algorithm for distinguishing primary cutaneous adnexal carcinomas from cutaneous metastases.

To navigate the complex problem of distinguishing between primary adnexal carcinomas and cutaneous metastases, we propose the following practical, stepwise algorithm. To facilitate the distinction between primary cutaneous adnexal carcinomas and cutaneous metastases, we propose a practical, stepwise diagnostic algorithm. This algorithm is based on a synthesis of the published dermatopathology literature, our institutional experience at a high-volume tertiary cancer center, and expert consensus. Although the algorithm has not yet undergone prospective multicenter validation, each of its individual components is supported by the evidence reviewed in the preceding sections. Future prospective validation across diverse practice settings will be important to establish its generalizability and clinical utility. A visual summary of the proposed algorithm is provided in Figure 1.

**Step 1: Clinical and Historical Assessment.** Begin with a thorough review of the patient’s age and sex, anatomic site of the lesion, and, most important, any history of a prior malignant neoplasm. Determine the temporal relationship (synchronous vs. metachronous) and the number and distribution of lesions. A solitary lesion in a patient without a prior malignant neoplasm favors a primary tumor, while multiple dermal nodules in a patient with known advanced cancer strongly favor metastasis.

**Step 2: Low-Power Histologic Evaluation.** Assess the architectural pattern on hematoxylin and eosin staining. Look for an epidermal connection, an in situ component, or a transition from a benign precursor, which strongly favors a primary adnexal origin. Conversely, a purely dermal/subcutaneous location, a “bottom-heavy” architecture, and intravascular tumor emboli favor metastasis.

**Step 3: High-Power Cytologic Evaluation.** Identify specific cytomorphologic features that suggest a particular lineage, such as clear cells, mucin production, signet ring cells, basaloid nesting, neuroendocrine chromatin, or sebaceous differentiation.

**Step 4: Initial IHC Panel (Primary Adnexal Screen)**. Employ a panel of IHC markers for primary adnexal carcinoma, including p63 (or p40), CK15, D2-40, and calretinin. Strong, diffuse positivity for these markers supports a primary cutaneous adnexal neoplasm. A negative result for all 4 markers is suggestive of metastasis and should prompt an organ-specific workup, although poorly differentiated primary adnexal carcinomas may occasionally show loss of these markers.

**Step 5: Lineage-Specific IHC Markers.** If the initial panel is negative or equivocal or if morphology strongly suggests metastasis, employ organ-specific markers based on the suspected primary tumor site: GATA3, TRPS1, and estrogen receptor for breast; TTF-1 and Napsin A for lung; CK20, SATB2, and CDX2 for colorectal; PAX8 for renal or Müllerian; NKX3.1 and prostate-specific antigen for prostate; and CK20 paranuclear dot-like pattern and INSM1 for Merkel cell carcinoma. For tumors with sebaceous morphology, adipophilin should be employed to confirm sebaceous differentiation, and SOX10 should be considered for tumors with myoepithelial or adenoid cystic features.

**Step 6: Molecular Testing.** In diagnostically ambiguous cases or when a specific fusion–driven adnexal tumor is suspected, utilize fluorescence in situ hybridization or next-generation sequencing to identify defining molecular alterations (e.g., *MYB*, *YAP1*, *CRTC1*, or *ETV6* fusions). Perform IHC or PCR studies for MMR genes on all sebaceous neoplasms to screen for Muir–Torre syndrome.

Limitations of the Proposed Algorithm. This algorithm is intended to serve as a practical diagnostic framework and may require adaptation based on institutional resources, clinical context, and specific differential diagnosis. Not every step will be necessary in all cases. For example, when morphologic features are characteristic of a particular entity, the diagnostic workup may be streamlined. Conversely, diagnostically challenging cases may require iterative application of multiple steps, additional ancillary studies, and multidisciplinary discussion. Furthermore, the algorithm assumes access to a comprehensive immunohistochemistry laboratory. Institutions with more limited resources may need to tailor immunohistochemical testing by prioritizing the most informative markers according to the clinical and morphologic context.

## 11. Conclusions

Distinguishing primary cutaneous adnexal carcinomas from cutaneous metastases of adenocarcinomas remains one of the greatest diagnostic challenges in dermatopathology because of their substantial clinicopathologic and immunophenotypic overlap. Morphologic evaluation alone is often insufficient. Instead, accurate diagnosis requires integration of clinical information, histopathologic findings, and a thoughtfully selected immunohistochemical panel. Markers such as p63/p40, CK15, D2-40, and calretinin provide valuable diagnostic information when interpreted in the appropriate morphologic and clinical context, while recognition of their limitations is essential to avoid diagnostic pitfalls. In most cases, this integrated approach allows for accurate classification of the lesion, which is more commonly a primary cutaneous adnexal neoplasm than a cutaneous metastasis.

The expanding repertoire of immunohistochemical markers, including SOX10, TRPS1, adipophilin, INSM1, and SATB2, has further improved the evaluation of diagnostically challenging cases. In parallel, advances in molecular pathology have transformed the classification of adnexal tumors by identifying recurrent gene fusions and characteristic mutational profiles that serve as highly specific diagnostic biomarkers. Although access to molecular testing remains variable, the growing availability of referral-based testing and immunohistochemical surrogate markers is making these advances increasingly accessible in routine practice.

As our understanding of the molecular pathogenesis of cutaneous adnexal neoplasms continues to evolve, the integration of conventional histopathology, immunohistochemistry, and molecular diagnostics will increasingly define the diagnostic standard. This multimodal approach promises greater diagnostic precision, more accurate tumor classification, and ultimately more informed therapeutic decision-making for patients with malignant cutaneous neoplasms.

## Figures and Tables

**Table 1:** Common Morphologic Patterns Seen in Both Primary Adnexal Carcinomas and Cutaneous Metastases.

Morphologic Pattern	Primary Adnexal Tumors with Pattern	Cutaneous Metastases with Pattern	Key Distinguishing Features
Clear cell	Sebaceous carcinoma, clear cell hidradenoma	Clear cell RCC	Primary adnexal tumor: epidermal connection, sebaceous lobules, PAX8−, CD10+
Mucinous	Primary mucinous (eccrine) carcinoma	Mucinous breast carcinoma, mucinous colorectal adenocarcinoma	Primary adnexal tumor: p63+ Breast: ER+, GATA3+ Colorectal: CDX2+, CK20+
Glandular/ductal	Apocrine carcinoma, eccrine carcinoma	Breast, lung, pancreatic adenocarcinoma	Primary adnexal tumor: p63+, CK15+ Breast: ER+, GATA3+ Lung: TTF-1+, Napsin A+
Basaloid/nested	Adenoid cystic carcinoma, spiradenocarcinoma	Small cell lung carcinoma, Merkel cell carcinoma	Small cell lung: TTF-1+, synaptophysin+ MCC: CK20 paranuclear dot-like pattern
Pagetoid intraepidermal	Primary EMPD, porocarcinoma	Secondary EMPD (colorectal, urothelial)	Primary EMPD: CK7+, GCDFP-15+ Colorectal: CDX2+, CK20+ Urothelial: uroplakin+
Signet ring cell	Signet ring cell hidradenoma	Gastric, lobular breast carcinoma	Gastric: CDX2+, CK20+ Lobular breast: GATA3+, E-cadherin loss

−, negative; +, positive; ER, estrogen receptor; MCC, Merkel cell carcinoma. Note: The immunophenotypes listed represent typical patterns and are not absolute. Exceptions occur in all categories, and results must be interpreted in the context of the full clinicopathologic picture. For example, triple-negative breast carcinomas may lack ER and GATA3 expression, and rare clear cell RCCs may show focal CK7 positivity.

**Table 2:** Key Molecular Alterations in Primary Cutaneous Adnexal Tumors and Their Extracutaneous Homologues.

Tumor Type	Molecular Alteration	Frequency	Homologous Extracutaneous Tumor
Adenoid cystic carcinoma	*MYB::NFIB* or *MYBL1::NFIB* fusion	73–83%	Salivary gland ACC
Poroma and porocarcinoma	*YAP1* fusion (e.g., *YAP1::MAML2*)	~88%	—
Hidradenoma and hidradenocarcinoma	*CRTC1::MAML2* or *CRTC3::MAML2* fusion	50–75%	Mucoepidermoid carcinoma (salivary gland)
Secretory carcinoma	*ETV6::NTRK3* fusion	~100%	Secretory carcinoma (breast, salivary gland)
Cylindroma	*CYLD* inactivation	~100%	—
Spiradenoma	*CYLD* inactivation or *ALPK1* mutation	~70% combined	—
Mixed tumor (chondroid syringoma)	*PLAG1* fusion	~33%	Pleomorphic adenoma (salivary gland)
Myoepithelioma	*EWSR1* or *FUS* fusion	~100%	Myoepithelioma (salivary gland, soft tissue)
Sebaceous tumors (Muir–Torre syndrome)	MMR loss (*MSH2*, *MSH6*), MSI-H	~15–20%	Lynch syndrome–associated visceral cancers

ACC, adenoid cystic carcinoma; MSI-H, microsatellite instability–high. Note: The frequency of *PLAG1* fusion in mixed tumors (chondroid syringomas) listed as ~33% may represent an underestimate; more recent literature suggests higher frequencies in classic mixed tumors, and this number should be interpreted with caution pending larger confirmatory studies.

## Data Availability

No new data were created.
